# Application of Multi-Criteria Decision Analysis Techniques for Informing Select Agent Designation and Decision Making

**DOI:** 10.3389/fbioe.2022.756586

**Published:** 2022-06-03

**Authors:** Segaran P. Pillai, Julia A. Fruetel, Kevin Anderson, Rebecca Levinson, Patricia Hernandez, Brandon Heimer, Stephen A. Morse

**Affiliations:** ^1^ Office of the Commissioner, Food and Drug Administration, U.S. Department of Health and Human Services, Silver Spring, MD, United States; ^2^ Sandia National Laboratory, U.S. Department of Energy, Livermore, CA, United States; ^3^ Science and Technology Directorate, U.S. Department of Homeland Security, Washington, DC, United States; ^4^ IHRC, Inc., Atlanta, GA, United States

**Keywords:** select agents and toxins, risk assesment, multi-criteria, logic tree analysis, biosecurity

## Abstract

The Centers for Disease Control and Prevention (CDC) Select Agent Program establishes a list of biological agents and toxins that potentially threaten public health and safety, the procedures governing the possession, utilization, and transfer of those agents, and training requirements for entities working with them. Every 2 years the Program reviews the select agent list, utilizing subject matter expert (SME) assessments to rank the agents. In this study, we explore the applicability of multi-criteria decision analysis (MCDA) techniques and logic tree analysis to support the CDC Select Agent Program biennial review process, applying the approach broadly to include non-select agents to evaluate its generality. We conducted a literature search for over 70 pathogens against 15 criteria for assessing public health and bioterrorism risk and documented the findings for archiving. The most prominent data gaps were found for aerosol stability and human infectious dose by inhalation and ingestion routes. Technical review of published data and associated scoring recommendations by pathogen-specific SMEs was found to be critical for accuracy, particularly for pathogens with very few known cases, or where proxy data (e.g., from animal models or similar organisms) were used to address data gaps. Analysis of results obtained from a two-dimensional plot of weighted scores for difficulty of attack (i.e., exposure and production criteria) vs. consequences of an attack (i.e., consequence and mitigation criteria) provided greater fidelity for understanding agent placement compared to a 1-to-n ranking and was used to define a region in the upper right-hand quadrant for identifying pathogens for consideration as select agents. A sensitivity analysis varied the numerical weights attributed to various properties of the pathogens to identify potential quantitative (x and y) thresholds for classifying select agents. The results indicate while there is some clustering of agent scores to suggest thresholds, there are still pathogens that score close to any threshold, suggesting that thresholding “by eye” may not be sufficient. The sensitivity analysis indicates quantitative thresholds are plausible, and there is good agreement of the analytical results with select agent designations. A second analytical approach that applied the data using a logic tree format to rule out pathogens for consideration as select agents arrived at similar conclusions.

## Introduction

In the 1990s, Congress recognized the need for regulations to ensure the biosafety and biosecurity of activities that involved the use of hazardous biological agents and toxins, and the facilities in which these activities occur. In 1996 Congress passed the Antiterrorism and Effective Death Penalty Act (Public Law 104-132, 1996); Section 511 of this Act addressed the heightened concern about the ease with which disease-causing agents could be obtained. The legislation directed the Department of Health and Human Services (HHS) to establish a list of biological agents and toxins (i.e., HHS select agents) that could potentially threaten public health and safety, establish procedures governing the transfer of those agents, and training requirements for entities working with them. This led to the promulgation of the Select Agent Regulation. HHS delegated the authority to administer this regulation to CDC (i.e., CDC Select Agent Program).

After the intentional release of anthrax spores through the U.S. mail in the fall of 2001, regulations to control these agents were enhanced (Morse, 2015). Congress significantly strengthened oversight of select agents with the passage of the Uniting and Strengthening America by Providing Appropriate Tools Required to Intercept and Obstruct Terrorism Act of 2001 (USA PATRIOT Act) (Public Law 107-56, 2001) and the Public Health Security and Bioterrorism Preparedness and Response Act of 2002 (Bioterrorism Act) (Public Law 107-188, 2002). The USA PATRIOT Act restricted who could have access to select agents, and the Bioterrorism Act created increased safeguards and security measures for select agents. The Bioterrorism Act required that the list of HHS select agents and toxins be reviewed and republished at least biennially, and that the HHS Secretary consider the following criteria in determining whether to include a biological agent or toxin on the list: the effect on human health of exposure to an agent or toxin; the degree of contagiousness of the agent and the methods by which the agent or toxin is transferred to humans; the availability and effectiveness of pharmacotherapies and immunizations to treat and prevent illnesses resulting from an agent or toxin; and any other criteria including the needs of children and other vulnerable populations that the HHS Secretary deems relevant (Public Law 107-188, 2002). The CDC Select Agent Program utilizes these and additional criteria (CDC, HHS, 2020) together with SME guidance to support the biennial review process.

To support the CDC Select Agent Program biennial review process, we developed and evaluated two risk-based analytical approaches for classifying bacteria and viruses as HHS select agents: a multi-criteria decision analysis (MCDA) framework and a decision support framework (DSF) in a logic tree format with a focus on agent properties and public health. Factors such as financial or opportunity cost were not considered. Previous efforts by the CDC Select Agent Program relied on SME assessments to rank the agents and have not applied the approach broadly to include non-select agent pathogens due to the additional burden placed on the SMEs. The analytical approaches presented here seek to provide a system engineering approach and decision analysis techniques for assessing bioterrorism risk, and to reduce the burden on SMEs by documenting the supporting data from the peer-reviewed literature in archivable data sheets. We applied the methodology broadly to evaluate the general applicability of the approach by including a variety of non-select agents in the assessment, such as Risk Group 3 and 4 pathogens (NIH, 2019), emerging pathogens, and former select agents that had been removed from the list by the Select Agent Program. The results of this initial assessment of such an approach for classifying select agents is presented here.

MCDA is a sub-discipline of operations research and is comprised of a set of methodological approaches that are well described (Keeney and Raiffa, 1993; Greco et al., 2005; Hwang and Yoon, 2012) and well-documented in the literature for conducting structured risk assessments (Linkov et al., 2006). MCDA offers a transparent method for conducting risk assessments that serve to quantify and communicate risk and support a risk management-based approach to decision making. This method allows for data uncertainty, can combine multiple information sources including those based upon expert judgement, and is simple in concept and amenable to a user-friendly software tool. Disadvantages of MCDA include those cited for qualitative methodologies, i.e., the lack of absolute measurements and potential for rank reversal (i.e., the possibility that adding a new option to, or removing an option from, a set of ranked options and redoing the analysis might change, or even reverse, the relative ordering of the other options, even though none of their attributes had been changed) (Cox et al., 2005). The use of MCDA for risk-based decision making has been described for environmental applications (Kiker et al., 2005; Steele et al., 2009), healthcare (Velasquez and Hester, 2013; Thokala et al., 2016), as well as emerging threats to animal and plant health (Cook and Proctor, 2007) and foodborne pathogens (Ruzante et al., 2010).

## Methods

### Analytical Framework

The three basic elements of an MCDA approach are (1) key values or criteria that form the basis for decision making, (2) the analytical framework that relates the key values and measures to the decision outputs, and (3) the underlying data and inputs that inform the scoring. To evaluate options against the key values, every criterion has a scoring scale based on linear ordinal values (e.g., 0–10), definitions for each numerical score, and an assigned weight. Options are scored on the basis of underlying data or SME input using the provided scoring definitions; weights are typically assigned through SME elicitation. The analytical framework describes the hierarchy for how the overall scores for each option are tallied based on the underlying scores; a common approach is to combine all the criteria and weights into a single score (A) by summing all the weighted numerical values (a_ij_,w_i_), where a_ij_ represents a criteria score and w_j_ is the criteria weighting value:

$$A=\;\overset{n}{\underset{j=1}{\sum }}aij•wj$$

Calculation of overall scores enables comparison of considered options through means such as rank order, where the options are ranked 1–n. Common applications of the MCDA technique are to select a single preferred outcome, which is typically the highest-ranking option; however, other output options are possible (Greco et al., 2005).

**FIGURE 1:**
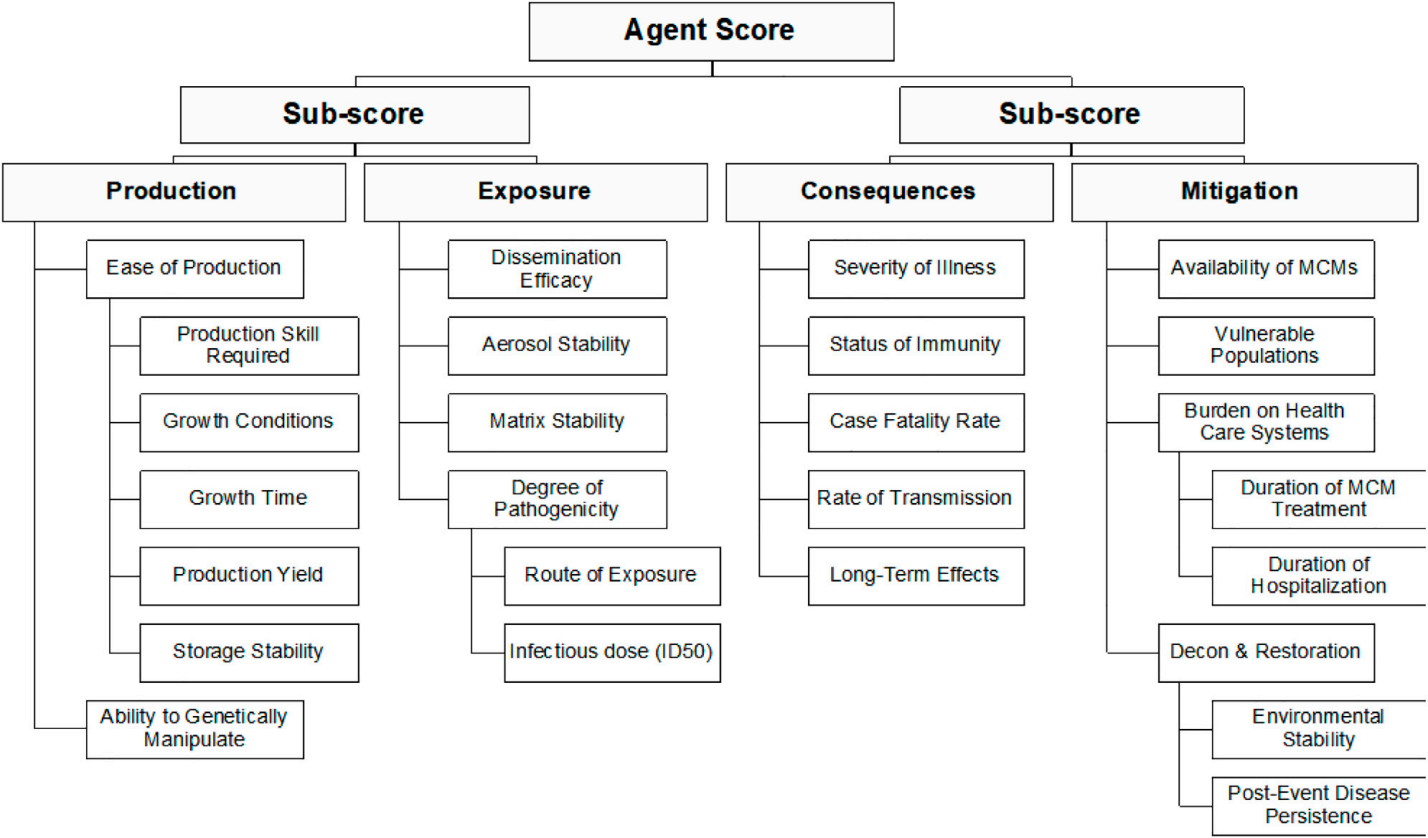
Summary of the criteria and hierarchy captured in the MCDA framework and fact sheets.

The criteria and hierarchy used are shown in Figure 1. This hierarchy includes the criteria set forth by the Bioterrorism Act and Select Agent Regulation (Public Law 107-188, 2002; CDC, HHS, 2020) and breaks down the agent score into key elements of bioterrorism risk: i.e., difficulty of a successful attack and consequence. These elements are further broken down into factors that measure the relative difficulty of agent production and population exposure for inhalation and ingestion scenarios, and the impact on human consequence and potential mitigation measures. Four criteria—Ease of Production, Degree of Pathogenicity, Burden on Health Care Systems, and Decon and Restoration—incorporate sub-criteria. For these criteria, scores are calculated as the average of the sub-scores. For example, the final score for the Ease of Production criteria is the average of the scores for Production Skill Required, Growth Conditions, Growth Time, Production Yield, and Storage Stability. Calculating the scores in this way assigns all the sub criteria of any given criterion equal weight.

For each criterion, a scoring scale of 0–10 was adopted. The scoring scale reflects relative concern as it pertains to the agent designation as a select agent, with 0 corresponding to lowest concern and 10 corresponding to highest concern. For simplicity, a linear scale was chosen for the initial evaluation; however, the scale can be non-linear. Definitions are provided for each of the criteria shown in Figure 1 and the even-numbered scoring options (0, 2, 4, 6, 8, and 10) (Supplementary Table S1). Although odd-numbered scores are allowed, no specific definitions are provided for them; however, odd numbered scores could account for options intermediate in concern between the two adjacent defined scores, based on SME judgement.

Two views of the data were explored for assessing the relative ranking of the agents: 1) a one-dimensional “risk ranking” whereby the total weighted sum for each agent is tallied and the agents are ranked from lowest to highest; and 2) a two-dimensional “risk style” plot whereby the weighted sum of the sub-scores for the “production” plus “exposure” branches of the hierarchy are plotted against the weighted sum of the sub-scores for the “consequences” plus “mitigation” branches of the hierarchy (see Figure 1). This plot is similar to standard risk plots of likelihood *versus* consequence.

To enable comparison of results using different weighting values, we utilized normalized scores, whereby the total or sub-total scores are normalized to those of a hypothetical “maximal agent” that would receive 10s for all the criteria scores.

### Agent Fact Sheets

To document the data used for scoring pathogens against the criteria shown in Figure 1, we developed fact sheets for 73 pathogens (Table 1); the list includes 36 HHS select agents, nine former HHS select agents, 21 Risk Group 3 and 4 pathogens, and four emerging pathogens. Five HHS select agents identified for removal from the current select agent list (*B. anthracis* Pasteur strain, *Brucella abortus*, *Brucella suis*, *Coxiella burnetii*, and *Rickettsia prowazekii*) were included as current select agents as their removal was not formally approved at the time of this study (CDC, HHS, 2020).

**TABLE 1:** HHS Select and non-select agents evaluated in this study.

Bacteria
** ** *Bacillus anthracis* *^a^* ^*,*^ *^b^*
** ** *Bacillus anthracis Pasteur strain* *^b^* ^*,*^ *^c^*
** ** *Bacillus cereus biovar anthracis* *^a^*
** ** *Bartonella*
** ** *Brucella abortus* *^b^* ^*,*^ *^c^*
** ** *Brucella melitensis* *^b^* ^*,*^ *^c^*
** ** *Brucella suis* *^b^* ^*,*^ *^c^*
** ** *Burkholderia mallei* *^a^* ^*,*^ *^b^*
** ** *Burkholderia pseudomallei* *^a^* ^*,*^ *^b^*
** ** *Clostridium botulinum* *^a^*
** ** *Coxiella burnetii* *^c^*
** ** *Francisella tularensis* *^a^*
** ** *Mycobacterium bovis*
** ** *Pasteurella multocida type B*
** ** *Rickettsia prowazekii* *^c^*
** ** *Rickettsia rickettsii* *^d^*
** ** *Vibrio cholerae*
** ** *Yersinia pestis* *^a^*
Fungi
** ** *Coccidioides immitis* ^d^
** ** *Coccidioides posadasii* ^d^
** ** *Histoplasma capsulatum*
Viruses
** **Chapare virus*^c^*
** **Chikungunya virus
** **Crimean Congo Hemorrhagic Fever virus (CCHFV)*^c^*
** **Dengue virus
** **Eastern Equine Encephalitis (EEE) virus*^c^*
** **Ebola virus^a^
** **Ebola virus (Reston and Bombali subtypes)
** **Flexal virus^d^
** **Guanarito virus*^c^*
** **Hantavirus (HFRS high path: Hantaan, Dobrava)
** **Hantavirus (HFRS low path: Seoul, Puumala)
** **Hantavirus (Sin Nombre)
** **Hantavirus (Andes)
** **Hendra virus*^b^* ^*,*^ *^c^*
** **Herpes B virus^d^
** **Human Immunodeficiency virus (HIV)
** **Human T Cell Lymphotropic virus (HTLV)
** **Influenza virus [Highly Pathogenic Avian Influenza (HPAI) H5N1]^e^
** **Influenza virus (H2N2; 1957–1968)
** **Influenza virus (1918 Spanish flu)*^c^*
** **Japanese encephalitis virus (JEV)^d^
** **Junin virus*^c^*
** **Kyasanur Forest Disease virus (KFDV)*^c^*
** **Lassa virus*^c^*
** **Lujo virus*^c^*
** **Lymphocytic choriomeningitis virus (LCMV)
** **Machupo virus*^c^*
** **Madariga virus (formerly EEE virus, S.A. Clade)^d^
** **Marburg virus^a^
** **Middle East Respiratory Syndrome Coronavirus (MERS-CoV)
** **Monkeypox virus*^c^*
** **Monkeypox virus (Congo Basin clade)^d^
** **Nipah virus*^b^* ^*,*^ *^c^*
** **Omsk virus*^c^*
** **Polio virus
** **Rift Valley Fever virus (RVFV)*^b^* ^*,*^ *^c^*
** **Sabia virus*^c^*
** **Severe Acute Respiratory Syndrome Coronavirus (SARS-1)*^c^*
** **Semliki Forest virus (SFV)
** **Simian Immunodeficiency virus (SIV)
** **Tick-Borne Encephalitis viruses (TBEV)*^c^*
** **TBEV (Central European subtype)^d^
** **Transmissible Spongiform Encephalopathies (TSE)
** ** *Variola* major^a^
** ** *Variola* minor*^c^*
** **Venezuelan Equine Encephalitis virus (VEEV 1AB and 1C)*^b^* ^*,*^ *^c^*
** **VEEV 1D and 1E (non 1AB and 1C subtypes)^d^
** **Vesicular Stomatis virus (VSV)
** **West Nile virus (WNV)
** **Whitewater Arroyo virus (WWAV)
** **Yellow Fever virus (YFV)
** **Zika virus

a Tier 1 select agent.

b Overlap select agent.

c Select agent.

d Removed from the select agent list (CDC, HHS, 2012).

e USDA select agent.

Development of the fact sheets utilized peer-reviewed open literature such as Medline, PubMed, Google Scholar and other unclassified data followed by extensive review by several SMEs who work with the specific pathogen. In cases where there were data gaps, data for similar organisms (e.g., same virus family) or appropriate animal models were captured to support scoring. When available, case fatality rates for the U. S. or countries with comparable levels of health care were used for scoring. In all cases, SME review was relied upon to provide expert judgement and concurrence on the best available data or basis for scoring. All agent fact sheets were reviewed by one or more SMEs identified by the CDC Select Agent Program. SMEs were asked to review the data provided on the fact sheets for accuracy and relevance, as well as the scores assigned to each data category. Comments received from the SMEs were incorporated into the sheets and scoring adjusted, as necessary.

### Criteria Weighting

To determine appropriate weighting of criteria, SMEs were asked to rank order the 15 criteria, where one describes the most important criterion and 15 describes the least important criterion for determining whether an agent should be a select agent. This approach was chosen to gauge the level of agreement among the SMEs; the average of the results obtained from 13 SMEs for each of the criteria is shown in Table 2. The results showed good agreement among SMEs in some areas; typically, at the two ends of the ranking spectrum. The SD ranged between 1.2 and 3.8. The highest agreement in ranking occurred for Degree of Pathogenicity, Availability of Medical Countermeasures, Rate of Transmission, and Dissemination Efficacy. Ease of Production and Ability to Genetically Manipulate also showed good agreement. The greatest disagreement in ranking occurred for Long-term Effects, Matrix Stability and Aerosol Stability. Case Fatality Rate and Severity of Illness also showed higher SD; however, 10 of 13 SMEs ranked Case Fatality Rate as number 1 and 11 of 13 SMEs ranked Severity of Illness in the top 3. The reasons for the disagreements were not explored further but may reflect differences in available information or exemplars used to inform individual ranking choices, among others (Hollingshead, 1996).

**TABLE 2:** Criteria rank ordering by SMEs and assignment to one of three weighting groups (A, B or C).

Criteria	Average ranking	SD	5/4/6 Weighting group assignment	3/3/9 Weighting group assignment
Case fatality rate	2.8	3.7	A	A
Degree of pathogenicity	3.0	1.2		
Severity of illness	3.2	3.6		
Rate of transmission	4.7	2.0		B
Availability of MCM	5.8	1.8		
Dissemination efficacy	6.2	2.2	B	
Ease of production	8.5	2.7		C
Burden on health care system	8.8	3.1		
Aerosol stability	8.9	3.3		
Matrix stability	10.3	3.5	C	
Status of immunity	10.4	3.0		
Long-term effects	11.2	3.8		
Decon and restoration	11.5	2.8		
Vulnerable populations	11.9	3.1		
Ability to genetically manipulate	12.0	2.5		

Because there was a lack of consensus on rank order, SMEs agreed to group similarly ranked criteria together and assign equal weighting values to the group. The results of the ranking exercise suggested that the criteria could be reasonably grouped into three categories for the assignment of weights, based on where the breaks occur in the average rankings, and for simplicity (Table 2). The average ranking results were used to group the top five criteria into Group A, the next four criteria into Group B, and the last six criteria into Group C (5/4/6 grouping). An alternative grouping was also evaluated whereby the top three criteria were placed in Group A, the next three in Group B, and the last nine into Group C (3/3/9 grouping). Both weighting options were explored to test the sensitivity of the results to how the groupings are specified. A baseline case of weighting values was tested where the ratio of the weights for Group A: Group B: Group C were set to be 3:2:1; specifically, a weighted value of three was defined for all criteria in group A, a weighted value of 2 for all criteria in group B, and a weighted value of 1 for all criteria in group C.

### Sensitivity Analysis

To study the sensitivity of the two-dimensional results to the choice of weights—both the numerical value assigned to the weights and the way the weight groups are defined (e.g., 5/4/6 and 3/3/9 groupings)—a sensitivity analysis was conducted where the numerical weighting values were randomly varied over a broad range (up to 100:1) while the agent scoring values were kept constant, creating thousands of weighting scenarios. For this analysis, the relative weights of Groups A, B and C are constrained as weight (Group A) ≥ weight (Group B) ≥ weight (Group C). Weights for Groups A and B were allowed to vary between one and 100, while the weight for Group C was fixed at one for all scenarios.

A secondary objective of the analysis was to investigate a quantitative basis for providing minimum scoring thresholds needed for designating a Select Agent. To evaluate this, a “select agent scoring region” was designated as the upper right quadrant in the two-dimensional plot formed by graphing difficulty of attack (weighted sum of exposure and production criteria) vs. consequences (weighted sum of consequence and mitigation criteria). This is the region where agents with the highest combined scores for Production, Exposure, Consequence and Mitigation would fall and thus they would represent the highest bioterrorism risk. To ensure the weighting scenario library does not include scenarios that allow clearly non-Select Agent scores to fall in the Select Agent region, we defined four simulated test agents that should not classify as select agents, and constrained the scenario set to only those scenarios where the test agents’ scores fall outside the select agent scoring region.

The four simulated test agents were assigned scores consistent with the following properties:

• Agent 1 is not a human pathogen
• Agent 2 does not cause severe disease and infection is non-fatal to humans
• Agent 3 does not cause infection in humans by inhalation or ingestion routes
• Agent 4 has characteristics of both Agents 2 and 3 (i.e., not infectious to humans by inhalation or ingestion, and does not cause severe disease nor mortality)

For the test agents, the targeted traits were scored low (typically 0) in the tool, while all other traits were scored as high as possible (10). In this way, the test agents serve as extreme cases of non-select agents; even though they may be perfect in all other attributes (e.g., very easy to produce in large quantities), they cannot be select agents because they don’t meet critical requirements. The scoring basis used to generate the test agents is shown in Table 3.

**TABLE 3:** Scoring basis used to generate four simulated test agents.

Criteria	Not human pathogen	Not infectious by inhalation or ingestion	Low severity and low CFR^a^	Not infectious^b^ and low severity and CFR
Ease of Production				
Production Skill	10	10	10	10
Growth Conditions	10	10	10	10
Growth Time	10	10	10	10
Production Yield	10	10	10	10
Storage Stability	10	10	10	10
Ability to Genetically Manipulate	10	10	10	10
Dissemination Efficacy	10	10	10	10
Aerosol Stability	0	0	10	0
Matrix Stability	0	0	10	0
Degree of Pathogenicity				
Route of Exposure	0	2	10	2
Infectious dose (ID50)	0	0	10	0
Severity of Illness	0	10	4	4
Status of Immunity	0	10	10	10
Case Fatality Rate	0	10	0	0
Rate of Transmission	0	10	10	10
Long-Term Effects	0	10	10	10
Availability of MCMs	0	10	0	0
Vulnerable Populations	0	10	10	10
Burden on Health Care				
Duration of MCM Treatment	0	10	0	0
Duration of Hospitalization	0	10	0	0
Decon and Restoration				
Environmental Stability	10	10	10	10
Post-Event Disease Persistence	0	10	10	10

a CFR, case fatality rate.

b By inhalation of ingestion.

The sensitivity analysis was run using scores for all 73 pathogens plus the four simulated test agents, with the select agent scoring region in the 2-D plot format initially defined by the lines x = 0.50 and y = 0.50 and then varied to optimize the size of the select agent scoring region to be as large as possible without artificially constraining the number of viable scenarios (i.e., test agent scores fall outside the Select Agent scoring region). We found that the thresholds of x = 0.36 and y = 0.56 for both weighting schemes studied (5/4/6 and 3/3/9) provided the optimal trade-off between permitting the largest number of viable scenarios while also allowing for a potentially permissive select agent window.

### Decision Support Framework

**FIGURE 2:**
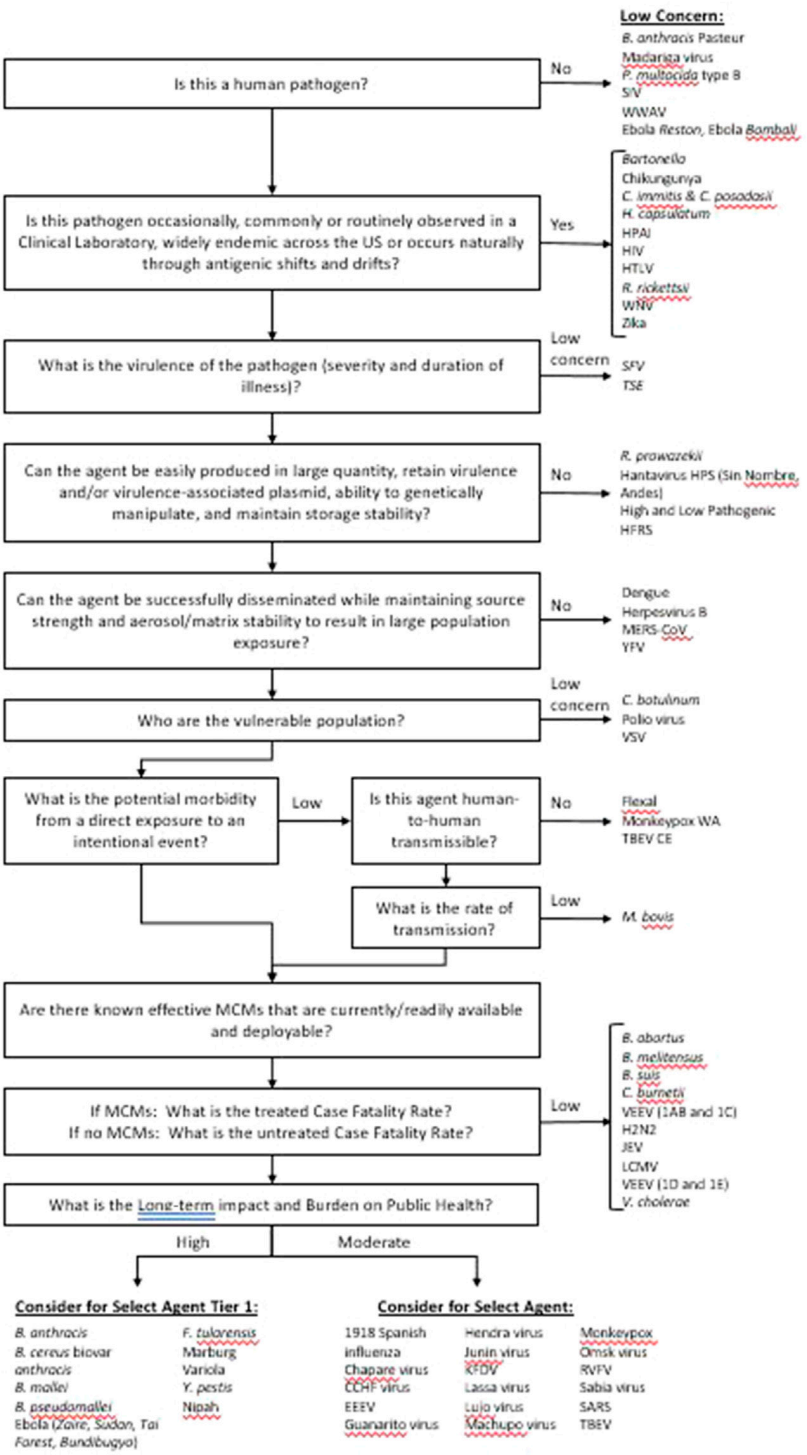
Decision Support Framework for assignments of select and non-select agents.

The DSF approach applies key criteria using a logic tree format to identify pathogens which may be of sufficiently low concern that they can be ruled out from consideration as a select agent, which is different from the MCDA criteria. The DSF is complementary and critical as a precursor to the analytical framework in order to avoid the possible unintended numerical equivalences from weighted sums. Knowledgeable adversaries will look at an entire proposed scenario to identify any “show-stoppers” that cannot be overcome, and toss out those that are infeasible, while seeking ways to overcome any undesirable characteristics of the remaining potentially feasible candidate scenarios. Frequently, the scenario selected for exploitation is not the one with the most advantages, but rather the one with the least disadvantages (in number and/or severity) so long as it meets the adversary’s basic criteria for desired outcome (often consequences or publicity). As a first step, the DSF considers the potential impact associated with regulating an agent versus the public health implications and practices. This avoids not regulating SARS-CoV-2 during the current pandemic to enable public health, health care practitioners, medical countermeasures and diagnostics developers, and scientists to perform their mission effectively. Using this approach, if a pathogen does not meet a threshold value for any one of the criteria set, it is deemed of low concern and thus is not considered a select agent. Those pathogens that exceed all criteria thresholds are considered for select agent status. Criteria include both risk-based (e.g., severity of illness and rate of transmission) and non-risk-based criteria such as clinical prevalence. The logic tree is shown schematically in Figure 2. SME judgment based on data captured in the agent fact sheets provided the basis for scoring. In general, criteria which received a score of zero or two typically served as a basis for a “low concern” qualitative assessment. In contrast to the MCDA approach, which uses a graded scoring system for ranking agents, the DSF approach can rule out an agent from select agent consideration using a single (low scoring) criterion. Many of the criteria overlap between the two approaches; however, there are key differences, such as the inclusion of clinical prevalence in the DSF approach.

## Results

### Data Gaps and Quality

When considering such a large number of micro-organisms across a broad range of attributes, data gaps and variability in data quality are inevitable. Data availability in the open literature tends to parallel scientific inquiry for the organism; for example, aerosol studies are more prevalent for pathogens known or suspected to be infectious by the aerosol route, and surface stability data are often more available for pathogens where fomite transmission is a concern. Overall, we found the most prominent data gaps were in aerosol stability and human infectious dose by inhalation and ingestion routes. For aerosol stability data, we typically used data for similar organisms (e.g., same virus family) as proxies, and infectious dose data for animal models was leveraged where available and needed to address human data gaps.

In the case of rare diseases where there is not a lot of human case data available, the data that are published tend to be for the severe cases reported by hospitals. Out of the 73 select and non-select agents examined (Table 1), six had fewer than 10 known cases: *B. cereus* biovar *anthracis* (zero cases), Chapare virus (one case) (Delgado et al., 2008), Flexal virus (two cases) (Archer and Rico Hesse, 2002), Lujo virus (5 cases) (Paweska et al., 2009), Sabia virus (four cases) (Barry et al., 1995; Lisieux et al., 1994) and Semliki Forest virus (one case) (Krauss, 2003; PHAC 2011). For four of these agents, the known cases were severe and the extent of mild or asymptomatic cases is largely unknown. Because it is difficult to extrapolate with confidence from a few cases to a potential large population exposure that may result from a bioterror attack, severity of illness and case fatality rates (CFRs) are poorly predicted from these data, and extrapolation tends to overestimate these parameters. To account for these factors, the Severity of Illness scoring definition was modified for a score of four to include “or <10 known cases” and the Case Fatality Rate definition for a score of two to include “or <10 known cases.” The changes in scoring definition had no effect on agents associated with mild disease (i.e., Flexal and Semliki Forest viruses), and significantly lowered scores for agents associated with rare severe disease (i.e., *B. cereus* biovar *anthracis*, Chapare virus, Lujo virus, Sabia virus). This approach was reviewed by the SMEs and felt to be consistent with current understanding of these organisms, given the gaps in data.

An additional challenge of applying the severity of illness and CFR criteria occurred for agents with a high proportion of asymptomatic or mild cases. CFRs for these agents may appear disproportionately high if they do not consider unreported asymptomatic and mild cases. To assess whether the CFR scores for agents with large proportions of asymptomatic or mild cases were too high, we reviewed the CFR scores for 15 agents and adjusted the scores for those with large asymptomatic or mild cases by multiplying the CFR by the percentage of severe cases (Table 4).

**TABLE 4:** Agents causing large numbers of asymptomatic or mild cases.

Agent	Asymptomatic or mild (%)	Case fatality rate		References
		Reported	Value used for scoring	
*M. bovis*	90% asymptomatic	5–6%	0.5–0.6%	Manjoor et al. (2011)
JEV	99% asymptomatic	20–30%	0.2–0.3%	CDC, (2019b)
WNV	>99% mild-moderate	10%	0.1%	Petersen and Marfin, (2002)
YFV	50–85% asymptomatic, 15–25% of symptomatic become severe	20–50%	0.4–6%	Monath, (2001)
Dengue virus	8–13% hospitalized	<1%	<1%	WHO, (2017)
*V. cholerae*	75% asymptomatic, 20% mild to moderate	1.3%	1.3%	CDC, (2018)
LCMV	33% asymptomatic	<1%	<1%	Peters, (2006)
*C. immitis*	60% asymptomatic or mild	0.1%	0.1%	DiCaudo, (2006)
				Huang et al. (2012)
*H. capsulatum*	“Most” asymptomatic or mild	4–8%	4–8%	Chu et al. (2006)
TBEV	70–95% asymptomatic	20–60% (Far Eastern); 6–8% (Siberian)	20–60% (Far Eastern); 6–8% (Siberian)	Gritsun et al, (2003)
RVFV	91–99% asymptomatic or mild	11–45%	<1%	Laughlin et al. (1979)
Omsk virus	50–70% mild-moderate	0.5–10%; 0.4–2.5%	0.5–10%; 0.4–2.5%	Růžek et al., (2010)
Lassa virus	80% mild	16.5–28%	16.5–28%	CDC, (2019c)
EEEV	95% mild	33%	33%	CDC, (2019a)
				Reed et al. (2007)
*B. melitensis*	64% mild	0.5%	0.5%	Buzgan et al. (2010)

Technical review of published data by SMEs expert in the specific pathogen was found to be important in general and in particular for pathogens with many data gaps, and where the use of proxy data for similar viruses led to high apparent total scores. Technical reviewers of limited published data available for White Water Arroyo virus, for example, concluded that it was questionable whether the virus causes human disease (Reignier et al., 2008; Zong et al., 2014). Similarly, scores for MERS-CoV based on similarity to other coronaviruses were adjusted upon review and updated to include recent data showing limited aerosol transmission likelihood (Kim et al., 2016; Arabi et al., 2017). While hantavirus is virulent when found in nature, technical reviewers indicated much lower virulence occurs when the virus is propagated in the laboratory (Lundkvist et al., 1997; McElroy et al., 2002; Nemirov et al., 2003; Safronetz et al., 2014; Prescott et al., 2017), suggesting much lower scoring is appropriate under public health consequences for this family of viruses.

### Unweighted Rankings

Initial inspection of the risk-ranking results (data not shown) indicated that, in general, the Tier 1 select agents were found at the top of the rank-ordered list (unweighted score 0.64–0.77), other select agents were located in the middle section (unweighted score 0.45–0.70), and non-select agents at the bottom (unweighted score 0.09–0.56). However, there were a few exceptions. For example, *R. prowazekii* (unweighted score 0.35) and *B. anthracis* Pasteur strain (unweighted score 0.31) fall near the bottom of the list. *C. botulinum* (unweighted score 0.44), a Tier 1 select agent, falls in the middle of the list; although an outlier among the Tier 1 agents, this is consistent with low scores in many consequence categories.

**FIGURE 3:**
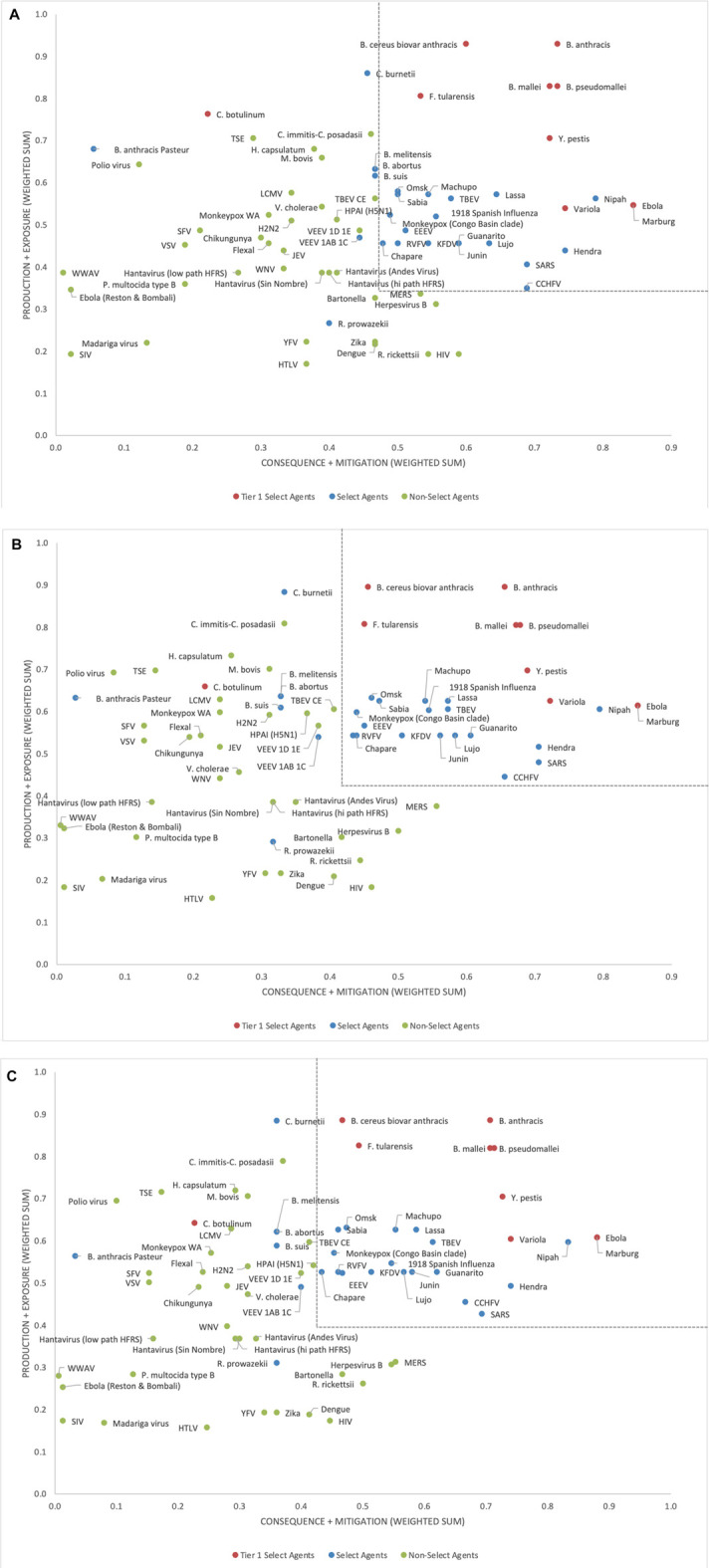
Two-dimensional plots of unweighted **(A)**, 5/4/6 grouping **(B)** and 3/3/9 grouping **(C)** normalized scoring results.

Analysis of the two-dimensional plot of the unweighted data (Figure 3A) shows similar groupings, where in this case the select agents generally fall into the upper right-hand quadrant of the plot, except for *R. prowazekii*, *B. anthracis* Pasteur strain and *C. botulinum*, which appear as outliers, and fall into other quadrants. When considering non-select agents, both the one- and two-dimensional plots indicated that, although there were general trends in the data that were consistent with current classifications, there are no sharp breaks in scoring that could serve as a basis or threshold for classifying select agents. Instead, the plots represent a continuum of scores. Any designation of a minimal score – whether as the total score in the one-dimensional plot, or as sub-scores corresponding to the x- and y-axes in the two-dimensional plot—will result in some exceptions to current classifications. For example, in the two-dimensional plot, if the lowest *x*-axis score for a select agent is designated as x = 0.48 and the y-axis score as y = 0.35 (Figure 3A), all select agents score in the select agent scoring region except *B. abortus*, *B. melitensis*, *B. suis*, *C. botulinum*, *C. burnetii, R. prowazekii*, *B. anthracis* Pasteur strain, and VEEV (1AB and 1C). All non-select agents score as non-select agents.

Because the two-dimensional plotted results provided greater fidelity for understanding agent placement compared to the one-dimensional risk ranking results, the one-dimensional approach was dropped, and the two-dimensional approach was pursued and is described in more detail below.

### Weighted Rankings

To evaluate the impact of applying criteria weights on the scoring results, initial numerical assignments for weights were chosen to be A:B:C = 3:2:1. Subsequent analysis of the impact of numerical weight assignments on the scoring assignments were evaluated more broadly (see below). Figure 3B shows the results of the weighted data in the two-dimensional format for the 5/4/6 grouping of criteria. Comparison of the results with the unweighted data indicate that in addition to the three select agents falling as outliers outside the upper right-hand quadrant (*R. prowazekii, B. anthracis* Pasteur strain and *C. botulinum*), several additional select agents (*B. abortus*, *B. melitensis*, *B. suis*, *C. burnetii* and VEEV (1AB and 1C) have pulled away slightly from the others. Also, a wider gap between select agents and non-select agents in the vertical dimension is observed with the weighted data, allowing for a potential threshold to fall between CCHFV and MERS-CoV. However, there was less of a gap in the data in the horizontal dimension, making the classification results for the borderline agents sensitive to the threshold choice. For example, if the lowest *x*-axis score for a select agent is designated as 0.41 and the y-axis score as 0.42 (dotted lines shown in Figure 3B), all select agents fall in the select agent scoring region except *B. abortus*, *B. melitensis*, *B. suis*, *C. botulinum*, *C. burnetii, R. prowazekii*, *B. anthracis* Pasteur strain, and VEEV (1AB and 1C). All non-select agents score as non-select agents.

The alternate 3/3/9 grouping for the criteria using A:B:C = 3:2:1 was analyzed to explore its impact on the results. The two-dimensional plot (Figure 3C) depicts the results of this analysis. If the lowest *x*-axis score for a select agent was designated as 0.43 and y-axis score as 0.40 (dotted lines in Figure 3C), all select agents reclassified as select agents except for *B. abortus*, *B. melitensis*, *B. suis*, *C. botulinum*, *C. burnetii*, *R. prowazekii*, *B. anthracis* Pasteur strain and VEEV (1AB and 1C). All non-select agents reclassified as non-select agents. These results were similar to those obtained with the 5/4/6 grouping.

The results from the weighted analysis using two different weighting schemes indicated that the designation of a “select agent region” in the two-dimensional plots, for the purposes of classifying select agents and distinguishing them from non-select agents, is possible but is sensitive to how the thresholds are set. Some select and non-select agent scores are quite close to the thresholding boundaries. To explore the sensitivity of the two-dimensional results to the choice of weights, and to investigate a more rigorous basis for defining the boundaries of the select agent region in the two-dimensional plot, we conducted a sensitivity analysis where the numerical weighting values were randomly varied over a broad range (up to 100:1) while the agent scoring values were kept constant, creating thousands of weighting scenarios. Similar Monte Carlo approaches for evaluating the sensitivity of weighting choices using rank order, additive sum models and multi-criteria decision models have been described previously and found helpful as an aid for supporting decision making (Butler et al., 1997).

### Sensitivity Analysis

**FIGURE 4:**
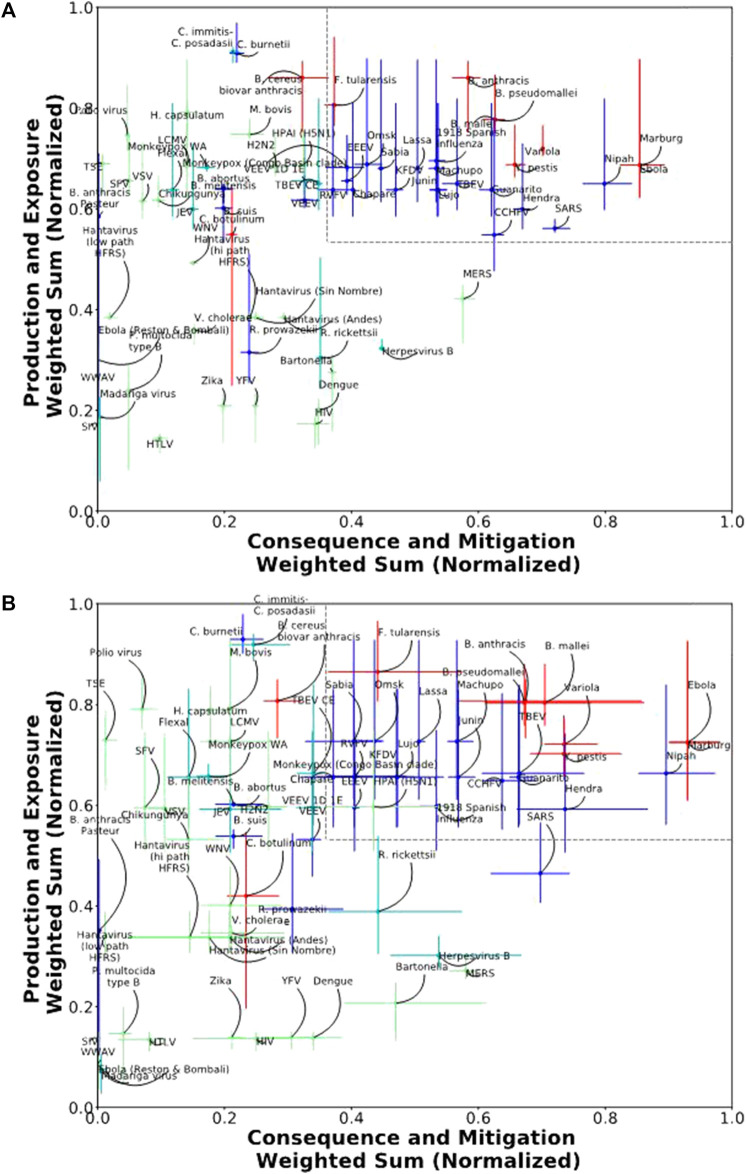
Two-dimensional plot of normalized median scores with error bars (5th and 95th percentiles) for **(A)** 5/4/6 grouping results and **(B)** 3/3/9 grouping results.

Using the threshold optimized to permit the largest number of scenarios while also allowing for a potentially permissive select agent window (x ≥ 0.36 and y ≥ 0.56) to define the select agent scoring region, we found that 97% of scenarios were viable for the 5/4/6 ABC grouping, and 96% of scenarios were viable for the 3/3/9 grouping. In this test, viable scenarios are those that exclude the four Test Agents from the select agent scoring region. The rejected scenarios differed slightly between the two groupings, but typically encompassed the lower scoring ratios (data not shown). Two-dimensional plots of the median scores with error bars indicating the 5th and 95th percentiles are shown for the 5/4/6 and 3/3/9 groupings in Figures 4A,B, respectively. The percentage of scenarios where each agent scored in the select agent scoring region for the 5/4/6 and 3/3/9 groupings are shown in Table 5. For the 5/4/6 grouping, the median scores for 20 agents landed within the select agent region for 100% of the scenarios and another six agents landed in the select agent region for >45% of the scenarios (Table 5). The drop-off was quite sharp after this, as only two more agents (*B. cereus* biovar *anthracis* [9.6%] and TBEV CE [0.6%]) spent any time at all in the select agent region.

**TABLE 5:** Percentage of scenarios in the sensitivity analysis where the agent score was within the select agent region as defined by thresholds x ≥ 0.36 and y ≥ 0.56.

Agent	5/4/6 Grouping	3/3/9 Grouping
1918 Spanish Influenza	100	67.2
*B. abortus*	0	0
*B. anthracis*	100	100
*B. anthracis* Pasteur	0	0
*B. cereus* biovar *anthracis*	9.6	0.4
*B. mallei*	100	100
*B. melitensis*	0	0
*B. pseudomallei*	100	100
*B. suis*	0	0
*C. botulinum*	0	0
*C. burnetii*	0	0
Ebola	100	100
EEEV	100	66.2
*F. tularensis*	89.3	97.2
Hendra	94.2	64.8
Junin	100	93.2
KFDV	100	93.2
Lassa	100	100
Lujo	100	84.3
Machupo	100	100
Marburg	100	100
MERS	0	0
Monkeypox (Congo Basin)	94.2	94
RVFV	69.7	93.2
Sabia	100	79.4
SARS	59.6	6.9
*Variola*	100	100
VEEV (1AB and 1C)	0	0
*Y. pestis*	100	100
*Bartonella*	0	0
*C. immitis-C. posadasii*	0	0
Chikungunya	0	0
Dengue	0	0
Ebola (Reston & Bombali)	0	0
Flexal virus	0	0
*H. capsulatum*	0	0
H2N2	0	0
Hantavirus (Andes)	0	0
Hantavirus (hi path HFRS)	0	0
Hantavirus (low path HFRS)	0	0
Hantavirus (Sin Nombre)	0	0
Herpes B virus	0	0
HIV	0	0
HPAI	0	67.0
HTLV	0	0
JEV	0	0
*M. bovis*	0	0
Madariga virus	0	0
Monkeypox WA	0	0
*P. multocida* type B	0	0
Polio virus	0	0
*R. rickettsii*	0	1.5
SFV	0	0
SIV	0	0
TBEV CE	0	0.1
TSE	0	0
*V. cholerae*	0	0
VEEV (1D and 1E)	0	0
VSV	0	0
WNV	0	0
WWAV	0	0
YFV	0	0
Zika	0	0

The sharp drop-off suggested a possible quantitative threshold for classifying select agents. A lenient threshold could be >0%; a more restrictive threshold could be >45%. If we consider the break point to be >5% of scenarios in the select agent region, then the classification results strongly parallel the two-dimensional 5/4/6 classification results using the 3:2:1 weighting.

Similar results were observed for the 3/3/9 grouping (Figure 4B). In this case, the median scores for 10 agents landed within the select agent region for 100% of the scenarios, and another 16 agents landed in the select agent region for >56% of scenarios (Table 5). The drop-off was quite sharp after this, as only five more agents spent any time at all in the select agent region (ranges between 0.1–6.9%). If we use a break point of >5% of scenarios in the select agent region, the classification results are similar to the two-dimensional 3/3/9 classification results using the 3:2:1 weighting except now *B. cereus* biovar *anthracis* is also excluded from the select agent region.

The results of the sensitivity analysis indicated that the reclassification of select and non-select agents using the two-dimensional method was robust over a broad range of weighting scenarios. In particular, the results supported the observations made using the 3:2:1 weighting assignment for both the 5/4/6 and 3/3/9 criteria groupings.

### Comparison of the MCDA and DSF approaches

To compare the results from the MCDA method with those using a different approach (logic tree) to identify select agents, the 73 select and non-select agents were evaluated using the DSF approach. The results are summarized in Figure 2. A comparison of results using this method with those obtained using the weighted two-dimensional MCDA approach indicated that both approaches classified *B. anthracis* Pasteur strain, *B. abortus*, *B. melitensis*, *B. suis*, *C. botulinum*, *C. burnetii*, *R. prowazekii* and VEEV (1AB and 1C) as non-select agents.

## Discussion

There are several reports in the open literature wherein authors describe approaches for evaluating pathogens for bioterrorism preparedness and other considerations. Rotz et al., 2002 utilized four high-level categories and 11 factors to evaluate 16 pathogens and toxins for designation as Category A, B or C agents. Cieslak et al., 2018 described a method using 12 attributes scored on a scale of zero to three for assessing the weapon potential of 33 pathogens and toxins. Casadevall and Pirofski, 2004, Casadevall and Pirofski, 2006 proposed using five criteria for assessing weapon potential, while MacIntyre et al. (2006), utilized 10 criteria for determining a risk priority score for six agents. Menrath et al., 2014 compared these and other approaches for evaluating and ranking biological agents. The aforementioned approaches faced many of the same challenges we did when we began this effort, including:

1) Lack of data to support measurements of critical criteria**.** Casadevall and Pirofski, 2004 noted this problem was a major limitation of their approach. As much as possible, we chose criteria and defined scoring options so that open-source data could be used for scoring. The areas where we found it most difficult to find data for all agents included infectious dose in humans and aerosol stability.
2) Reliance on SMEs to score agents. Several approaches relied heavily on SMEs for scoring. For example, Cieslak et al., 2018 relied on SMEs affiliated with the biodefense establishment of member nations of NATO’s Biological Medical Advisory Panel to score all the parameters for each organism. This likely contributed to the observed spread in scores, as it is challenging to find SMEs with expertise in all the factors assessed for an agent. For our approach, we initially scored agents using data from literature searches, then vetted the data and scoring with SMEs. We had more than one SME review each fact sheet, enabling each SME to review those areas where they have the most expertise.
3) Limited assessment scale (typically 0–3) with generalized scoring definitions (e.g., low/med/high). This factor, combined with a heavy reliance on SMEs, can result in widespread scores, as was observed by Cieslak et al., 2018. Our approach provided specific definitions for each scoring choice, literature data to inform scoring and SME verification of the resulting scores.
4) Use of criteria which are very difficult to accurately measure. Examples from the literature include public perception (Rotz et al., 2002) and a “terror factor” (Casadevall and Pirofski, 2004). We did not include these as criteria, as they are hard to predict, difficult to quantify and will vary over time. Any pathogen, if used to intentionally expose a population as part of a bioterrorism plot, is likely to generate some level of terror, disruption or public perceptions of fear.

In addition, the Select Agent biennial review process has often become highly contentious, as reflected in the often highly adverse reactions to efforts to remove certain pathogens from the list of regulated agents (Franz, 2015). A systematic approach as described herein offers the opportunity to provide substantial justification for such requests. The criteria we employed in this analysis are based on those identified in the Federal Registry Notice of Proposed Rule Making that the HHS Secretary should consider in determining whether to include a biological agent or toxin on the list (Public Law 107-188, 2002; CDC, HHS, 2020). Our goal for this study is to develop a robust and reproducible methodology for evaluating pathogens of concern for the establishment of a select agent list. As such we considered additional criterial to ensure it was a robust and to add additional fidelity to the process. Comparison of these criteria with other published methods shows that many of them overlap, such as morbidity and mortality, route of exposure, environmental stability, transmissibility, ease of production, availability of MCMs, etc. We included the status of immunity, an important host consideration pointed out by Casadevall and Relman, 2010.

In addition to the choice of criteria, the focus on bioterrorism scenarios (i.e., aerosol or food-based attacks affecting a large segment of the general population) is embodied in the scoring scales—for example, sexually transmitted pathogens rank low because aerosol and ingestion transmission routes are scored higher than those that are sexually transmitted. Also, pathogens that cause severe disease only in the presence of co-morbidities would rank low for Severity of Illness in the general population. Common pathogens causing mild illness and where there are treatments readily available may be unlikely to necessitate calling up a large-scale public health response.

Criteria we did not consider include public perception or terror factor, availability of agent and ease of detection, surveillance and laboratory diagnostic factors. Although we did not include incubation period as a specific criterion, we did take this factor into account as part of scoring Severity of Illness; i.e., diseases with long incubation periods (e.g., months to years) were considered chronic diseases and thus were scored lower than acute diseases. The fraction of symptomatic to non-symptomatic infections was considered as part of scoring CFR and severity of illness.

The results obtained using SME derived weights and the two-dimensional MCDA approach were tested against the current Select Agent Program designation and recent program decisions, and showed a high level of agreement, providing confidence that as new agents are considered, their terrorism risk can be assessed quickly and effectively using this method. The MCDA methodology reproduced all current select agent designations and all delisted select agents (Table 1) except for VEEV (1AB and 1C), which is currently a select agent but has been proposed for removal (CDC, HHS, 2020). In particular, the results are consistent with the 2020 proposal by the CDC Select Agent Program to remove *B. abortus*, *B. melitensis*, *B. suis*, *B. anthracis* Pasteur strain, *C. burnetii*, *R. prowazekii* (CDC, HHS, 2020) and confirms earlier decisions to remove *C. immitis*, *C. posadasii*, *R. rickettsii*, Flexal virus, Herpes B virus, Japanese encephalitis virus, Madariga virus, Monkeypox virus (Congo Basin clade), TBEV (Central European subtype) and VEEV (non 1AB and 1C subtypes) (CDC, HHS, 2012). The DSF also reproduces all current select agent designations, all delisted select agents (Table 1) and proposed select agents for removal including – VEEV (1AB and 1C). Both approaches confirm the exclusion of all non-select agents evaluated.

Two additional agents deserve specific mention. Highly Pathogenic Avian Influenza H5N1 (HPAI), a non-select HHS agent, received a score using the 3/3/9 criteria grouping with 3:2:1 weighting that placed it close to the threshold. However, it was classified as a non-select agent using the 5/4/6 weighting and the DSF approach. Although not an HHS select agent, HPAI remains a US Department of Agriculture select agent. *C. botulinum* is classified as a Tier 1 select agent (Bhattacharjee, 2011) because of the lethality of botulinum toxins; however, neither method evaluated here classified the organism as a select agent. *C. botulinum* spores are ubiquitous (Smith, 1978) and infections with this bacterium are rare (Werner et al., 2000). Analogous to the situations with ricin and castor beans and abrin and jequirity seeds, there appears to be little justification for keeping *C. botulinum* as a Tier 1 select agent.

Application of the methodology across a large and diverse pathogen set, while helping to demonstrate the robustness of the approach, highlighted the challenge of how to handle data gaps for many pathogens. The use of proxies and other assumptions at times artificially elevated some pathogens, requiring SME review of the data and discussions on how to account for the uncertainties in the data. Thus, we found the methodology also useful for identifying those parameters and pathogens where more data are needed, to help with prioritizing future research studies.

## Conclusion

The goal of this effort was to explore the use of MCDA and logic tree approaches for supporting Select Agent Program decision making. We found that the two methods show strong promise. The two-dimensional MCDA approach provided a risk-informed assessment that implements the Select Agent Program’s decision criteria and its focus on bioterrorism scenarios with the potential for large-scale public health consequences. The DSF is a complementary approach to classifying select agents and provides additional insight into the factors that influence decision making. The two methods represent different ends of a spectrum for using criteria thresholding to identify select agents: the MCDA approach applies thresholds to two sub-scores after considering the criteria shown in Figure 1, while the DSF approach applies thresholds at the single criterion level for nine criteria. Applying weights using the MCDA approach can be used to fine-tune the effective number of criteria used to identify a threshold.

Although the two-dimensional MCDA data did not present clear data breaks for establishing thresholds, we demonstrated an analytical approach for designating quantitative thresholds through the use of simulated test agents over a broad range of weighting scenarios. In general, we found that the Tier 1 agents (plus Nipah virus and minus *C. botulinum*) consistently ranked above the other agents. One might argue that it is logical to assume that these agents represented the highest risk of public health consequences and thus constituted a special group of pathogens that should be regulated. Below the Tier 1 agents is a second group of agents that pose a lower risk; this group included mostly select agents, although there are some non-select agents as well. Below that is a third group of agents that poses the lowest risk; this group is mostly non-select agents and includes some delisted select agents. One can argue that this lowest group should not require controls such as those required by the select agent regulations. The need for controls for the middle group of agents is then an area for further investigation. Casadevall and Relman, 2010 contended that the Select Agent Program should restrict the number of select agents to a very few, as that enables the others to be used for research. Exclusion of this middle group of agents from the regulations would be consistent with that viewpoint. It should be noted that these results represent the data at the time of scoring, but the methodology, developed to support the biennial review, allows for updates to the scores as new data emerges.

## Supplementary Material

Supplementary Table S1 Criteria scoring definitions.
