# Supplementary material for: Application of Multi-Criteria Decision Analysis Techniques for Informing Select Agent Designation and Decision Making
Source: Front Bioeng Biotechnol. 2022 Jun 3;10:756586. doi: 10.3389/fbioe.2022.756586 (PMC9204104; doi:10.3389/fbioe.2022.756586)
Supplement: Supplementary file 1 [file Table1.docx]

**Supplementary Table 1. Criteria Scoring Definitions**

| **PRODUCTION** |
| --- |
| **Ease of Production –** The ease of producing agent in the laboratory as measured by the skill required, availability of growth media and equipment, time required, yield and storage stability. |
| **Production Skill Required –** The level of training and agent-specific expertise needed to produce the agent and maintain pathogenicity: |
| \| 0 \| Difficult to produce \| \| --- \| --- \| \| 2 \| Expert-level training and agent specific experience \| \| 4 \| Expert-level training with similar organisms \| \| 6 \| Proficient in tissue culture and/or expert in aseptic technique \| \| 8 \| Basic microbiology training \| \| 10 \| Untrained \| |
| **Growth Conditions –** The availability of growth media, culture and/or equipment required to successfully grow the agent: |
| \| 0 \| No known cell lines available \| \| --- \| --- \| \| 2 \| Virus: Special post processing required \| \| 4 \| Virus: Special cell line required. Bacteria: Must be grown *in ovo* or *in vivo* \| \| 6 \| Virus: Common cell line required (e.g., Vero E6). Bacteria: Requires cell line or anaerobic conditions \| \| 8 \| Bacteria: Only grown in a single, complex broth or requires additional processing \| \| 10 \| Bacteria: Can be grown in common broths \| |
| **Growth Time –** The length of time to produce the agent based on growth characteristics of the agent: |
| \| 0 \| >1 month \| \| --- \| --- \| \| 2 \| 14-28 days \| \| 4 \| 10-13 days \| \| 6 \| 7-9 days \| \| 8 \| 3-6 days \| \| 10 \| 2 days or less \| |
| **Production Yield –** Highest concentration (pfu or cfu/mL) achieved by experts using optimal production methods: |
| \| 0 \| <10^2^ per mL \| \| --- \| --- \| \| 2 \| 10^2^-10^3^ per mL \| \| 4 \| 10^4^-10^5^ per mL \| \| 6 \| 10^6^-10^7^ per mL \| \| 8 \| 10^8^-10^10^ per mL \| \| 10 \| >10^10^ per mL \| |
| **Storage Stability –** The amount of agent lost during storage at 4^o^C: |
| \| 0 \| >1 log loss/day \| \| --- \| --- \| \| 2 \| 1 log loss/day \| \| 4 \| 1 log loss/week \| \| 6 \| 1 log loss/month \| \| 8 \| 1 log loss/year \| \| 10 \| <1 log loss/year \| |
| **Ability to Genetically Manipulate or Alter –** The degree of difficulty of the techniques required to create a more virulent, transmissible, environmentally stable or countermeasure-resistant strain: |
| \| 0 \| No known method to genetically manipulate and maintain pathogenicity \| \| --- \| --- \| \| 2 \| Very difficult (e.g., negative strand RNA viruses) \| \| 4 \| Highly difficult (e.g., positive strand RNA viruses, gene reassortment or reverse genetics available) \| \| 6 \| Moderately difficult (e.g., DNA viruses and intracellular bacteria) \| \| 8 \| Low difficulty (e.g., plasmid insertion for bacteria) \| \| 10 \| No directed genetic manipulation required (e.g., can use selection for antibiotic resistance) \| |
| **EXPOSURE** |
| **Dissemination Efficacy –** The ability of the agent to be disseminated effectively while maintaining viability and infectivity (aerosol or in water or food): |
| \| 0 \| Not able to be effectively disseminated \| \| --- \| --- \| \| 2 \| Low (e.g., enveloped viruses other than pox) \| \| 4 \| Low to moderate (e.g., gram negative bacteria) \| \| 6 \| Moderate (e.g., pox viruses, non-enveloped viruses) \| \| 8 \| Moderate to high (e.g., gram positive bacteria) \| \| 10 \| High (e.g., spore formers) \| |
| **Aerosol Stability** – The extent to which the agent is stable and infectious as an aerosol after dissemination (does not include dissemination losses): |
| \| 0 \| Very unstable (e.g., >20% loss/min at 25^o^C, 50% RH); or not infectious by inhalation \| \| --- \| --- \| \| 2 \| Relatively unstable (e.g., 11-20% loss/min at 25^o^C, 50% RH) \| \| 4 \| Low stability as an aerosol (e.g., 6-10% loss/min at 25^o^C, 50% RH) \| \| 6 \| Moderately stable as an aerosol (e.g., 1-5% loss/min at 25^o^C, 50% RH) \| \| 8 \| Very stable as an aerosol (e.g., 0.1-1% loss/min at 25^o^C, 50% RH) \| \| 10 \| Extremely stable as an aerosol (e.g., <0.1% loss/min at 25^o^C, 50% RH) \| |
| **Matrix Stability** – The extent to which the agent is stable and infectious in matrices such as milk, water and/or food over time: |
| \| 0 \| Agent decays immediately upon dissemination; or not infectious by ingestion \| \| --- \| --- \| \| 2 \| Stable in matrix for minutes to hours \| \| 4 \| Stable in matrix for hours to days \| \| 6 \| Stable in matrix for days to weeks \| \| 8 \| Stable in matrix for weeks to months \| \| 10 \| Stable in matrix for months to years \| |
| **Degree of Pathogenicity –** The ability of the agent to cause disease as measured by route of exposure and infectious dose. |
| **Route of Exposure –** The routes in which the disease is infectious to humans: |
| \| 0 \| None \| \| --- \| --- \| \| 2 \| Direct contact, cutaneous or vector \| \| 4 \| Ingestion \| \| 6 \| Inhalation \| \| 8 \| 2 different routes \| \| 10 \| 3 different routes \| |
| **Infectious dose (ID_50_) –** The dose or amount of agent (in cfu or pfu as appropriate) required to infect 50% of a healthy adult human population by inhalation or ingestion: |
| \| 0 \| Not infectious by inhalation or ingestion \| \| --- \| --- \| \| 2 \| >10,000 \| \| 4 \| 1000-10,000 \| \| 6 \| 100-1000 \| \| 8 \| 10-100 \| \| 10 \| 1-10 \| |
| **CONSEQUENCES** |
| **Severity of Illness** – The severity of the symptoms from acute illness: |
| \| 0 \| Little or no symptoms \| \| --- \| --- \| \| 2 \| Mild symptoms; resolves with rest at home \| \| 4 \| Requires bed rest and symptomatic treatment; or <10 known cases \| \| 6 \| Requires bed rest and a small subset of infected individuals require hospitalization (e.g., <25%) \| \| 8 \| Majority of infected individuals require hospitalization \| \| 10 \| All infected individuals require hospitalization with severe disease \| |
| **Status of Immunity** – The extent to which the population may have immunity to the disease due to previous exposure or vaccination: |
| \| 0 \| Close to 100% \| \| --- \| --- \| \| 2 \| Majority (>80%) of population have immunity \| \| 4 \| Significant portion (20-80%) of population have immunity \| \| 6 \| Previous vaccines may have reduced impact \| \| 8 \| Small subset (<5%) have immunity (e.g., at-risk workers, military) \| \| 10 \| No presumed immunity to agent in population \| |
| **Case Fatality Rate** – The number of deaths from the disease per 100 diagnosed cases (or number of known cases): |
| \| 0 \| Close to 0% \| \| --- \| --- \| \| 2 \| 1-9%; or <10 known cases \| \| 4 \| 10-29% \| \| 6 \| 30-39% \| \| 8 \| 40-49% \| \| 10 \| 50-100% \| |
| **Rate of Transmission** – The extent to which the disease can be transmitted from one human to another: |
| \| 0 \| Non-communicable and non-transmissible \| \| --- \| --- \| \| 2 \| Rare person-to-person transmission \| \| 4 \| R_o_ < 1 \| \| 6 \| 1 ≤ R_o_ < 2 \| \| 8 \| 2 ≤ R_o_ < 4 \| \| 10 \| R_o_ ≥ 4 \| |
| **Long-Term Effects** – The extent to which a significant portion of the affected population may incur disability or require additional medical care beyond treatment for acute illness (sequelae): |
| \| 0 \| None and/or unknown due to too few cases \| \| --- \| --- \| \| 2 \| Little to no long-term effects \| \| 4 \| No functional effects, but follow-up medical care required \| \| 6 \| Persists to a chronic stage for years and/or acute disease can reoccur \| \| 8 \| Long-term functional impacts (e.g., some vision loss, neurological) \| \| 10 \| Some level of permanent incapacitation, requiring long-terms care (e.g., complete blindness, limb loss) \| |
| **MITIGATIONS** |
| **Availability of Medical Countermeasures** – The availability of efficacious medical treatments/countermeasures and extent to which they can be rapidly deployed in response to a public health emergency: |
| \| 0 \| No MCMs required or would be deployed due to low morbidity and mortality \| \| --- \| --- \| \| 2 \| Widely available and easy to deploy efficiently (e.g. oral tablets/ capsules) \| \| 4 \| Widely available but difficult to deploy efficiently (e.g. intravenous-only drugs) or lacks efficacy \| \| 6 \| Approved treatment or vaccine available in limited quantities \| \| 8 \| Experimental treatment in development (e.g., EUA required) or only approved outside the US \| \| 10 \| No treatment beyond supportive care available \| |
| **Vulnerable Populations** – The portion of the population susceptible to a more severe form and/or complications from the disease: |
| \| 0 \| None \| \| --- \| --- \| \| 2 \| Small subset or group (e.g., rare genetic disorder) \| \| 4 \| Only immunocompromised \| \| 6 \| Immunocompromised and pregnant females \| \| 8 \| Immunocompromised, pregnant females and children or elderly \| \| 10 \| All \| |
| **Burden on Public Health Care Systems** – The potential burden to public health during and after an event, as measured by duration of medical countermeasure treatment and duration of hospitalization. |
| **Duration of MCM Treatment** – The length of time for medical countermeasure treatment required for a significant portion of the affected population: |
| \| 0 \| 0-6 days \| \| --- \| --- \| \| 2 \| 7-14 days \| \| 4 \| 15-21 days \| \| 6 \| 22-28 days \| \| 8 \| 29-35 days \| \| 10 \| >36 days \| |
| **Duration of Hospitalization** – The type and duration of medical care needed for a significant portion of the affected population to recover from the disease: |
| \| 0 \| No hospitalization required \| \| --- \| --- \| \| 2 \| Outpatient level care required with some hospitalization \| \| 4 \| Hospitalization typically required for <1 week \| \| 6 \| Hospitalization required between 1 week to 1 month \| \| 8 \| Hospitalization required for >1 month \| \| 10 \| Hospital isolation ward required \| |
| **Decontamination and Restoration** – The potential extent remediation efforts are needed due to agent persistence in the environment and population, as measured by environmental stability and post-event disease persistence in the population. |
| **Environmental Stability** – The extent to which the agent is stable in the environment (outside the host) in matrices such as soil and dried on surfaces: |
| \| 0 \| Agent decays immediately upon dissemination \| \| --- \| --- \| \| 2 \| Agent persists in indoor environments for minutes to hours \| \| 4 \| Agent persists in indoor environments for days to weeks \| \| 6 \| Agent persists in indoor environments for months to years or outdoors for hours to days \| \| 8 \| Agent persists in outdoor environments for weeks to months \| \| 10 \| Agent persists in outdoor environments for > 1 year \| |
| **Post-Event Disease Persistence** – The means by which the disease can persist in the population following an event: |
| \| 0 \| No persistence \| \| --- \| --- \| \| 2 \| Host to human transmission via vectors and/or wild animals \| \| 4 \| Host to human transmission via domestic animals \| \| 6 \| Environmental exposure (e.g., cutaneous) due to persistence in environment \| \| 8 \| Patient can transmit disease while pre- or post-symptomatic \| \| 10 \| Patient can transmit disease for a prolonged period of time \| |
